# True Colonic Melanosis: An Interesting Phenotypic Variation of Neurocristopathy

**DOI:** 10.1093/jcag/gwab018

**Published:** 2021-06-18

**Authors:** Mahum Nadeem, Lewis A Hassell, Hussein Bitar, Ijlal Akbar Ali

**Affiliations:** Internal Medicine, Oklahoma University Health Sciences Center, Oklahoma City, Oklahoma, USA; Department of Pathology, Oklahoma University Health Sciences Center, Oklahoma City, Oklahoma, USA; Department of Gastroenterology, Oklahoma University Health Sciences Center, Oklahoma City, Oklahoma, USA; Department of Gastroenterology, Oklahoma University Health Sciences Center, Oklahoma City, Oklahoma, USA

Pluripotent neural crest cells arise from the ectodermal layer of the embryo. These migrate and differentiate into array of cells varying from melanocytes to bone. Defect in the development or migration of these cells can cause a variety of phenotypic variations falling under an umbrella of neurocristopathy (1). Melanosis Coli is one of these benign neurocristopathies which involves proliferation of melanocytes in colon resulting in melanin deposition.

**Figure 1.**
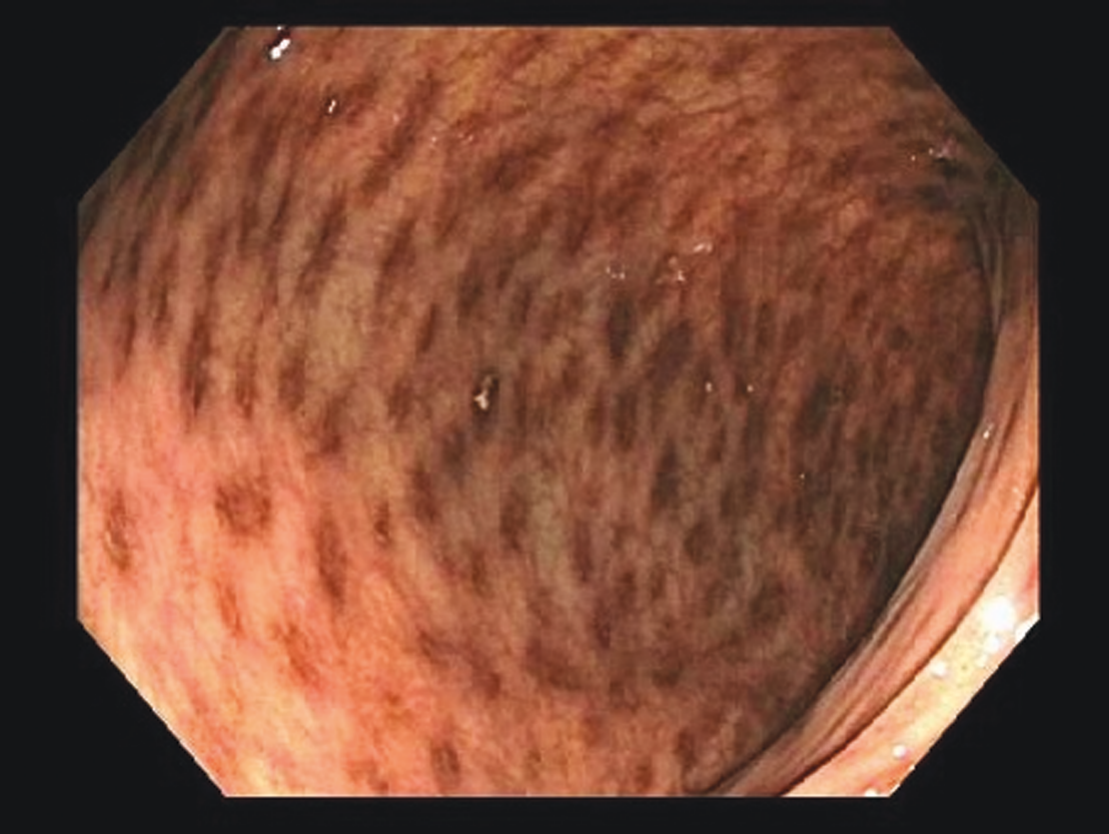
Endoscopic view of the descending colon showing patchy black pigmentation.

**Figure 2.**
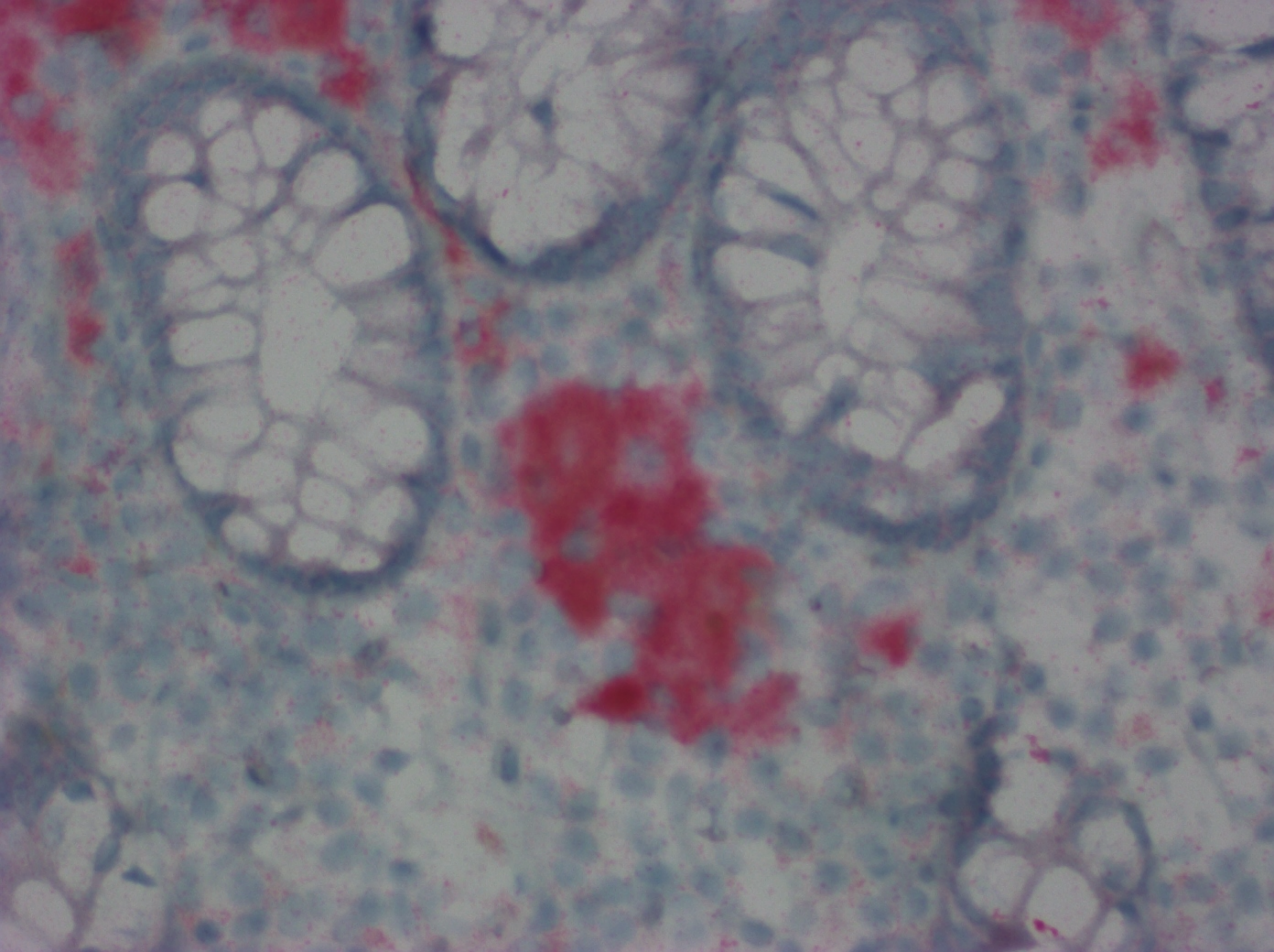
Immunohistochemical staining with MelanA after melanin bleach treatment demonstrates strong positive cytoplasmic staining in the pigmented cells. (400×).

**Figure 3:**
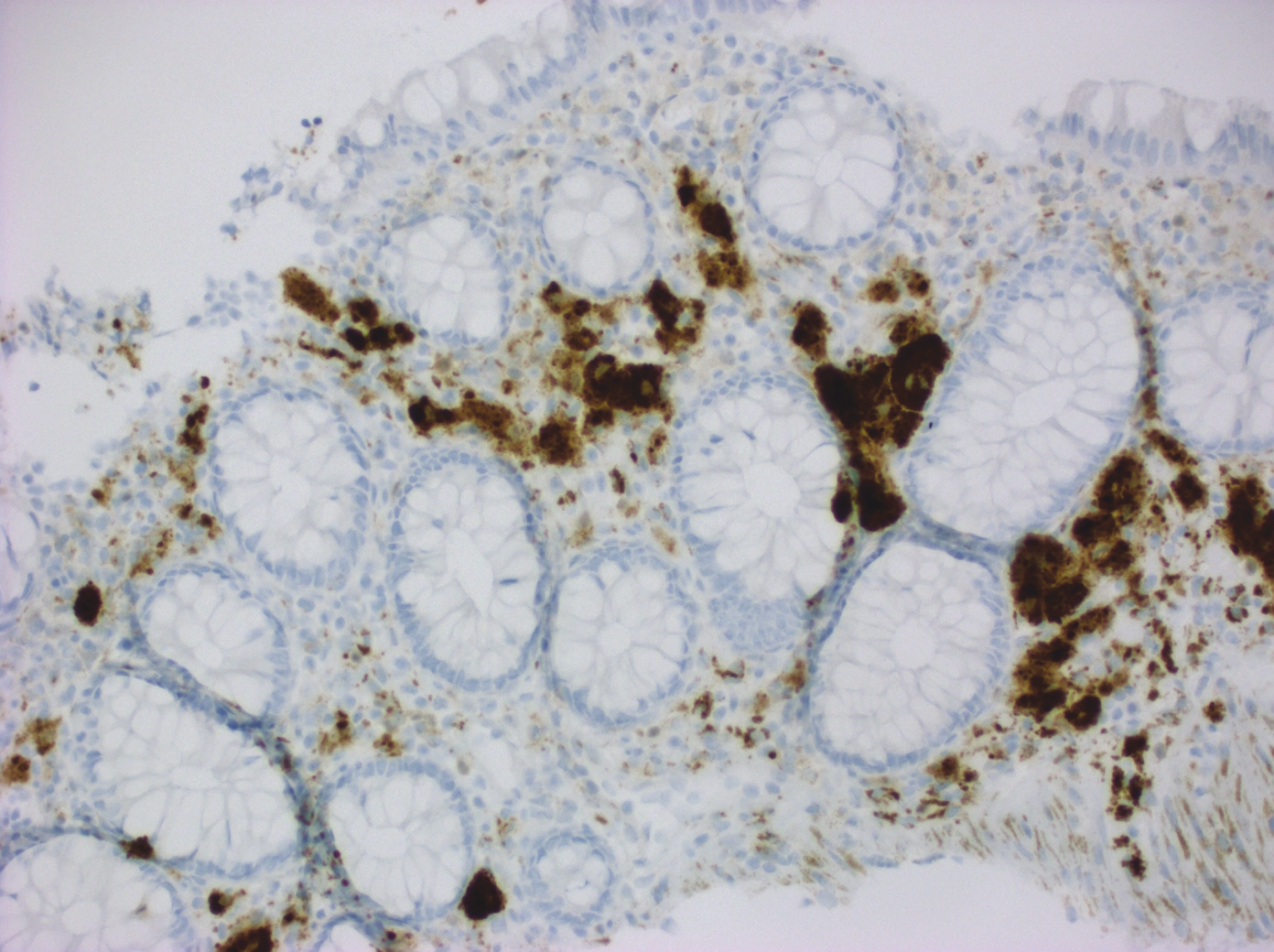
Immunohistochemical staining with Microphthalmia Transcription Factor (MiTF) stains the large pigmented cells positively. (200x)

**Figure 4:**
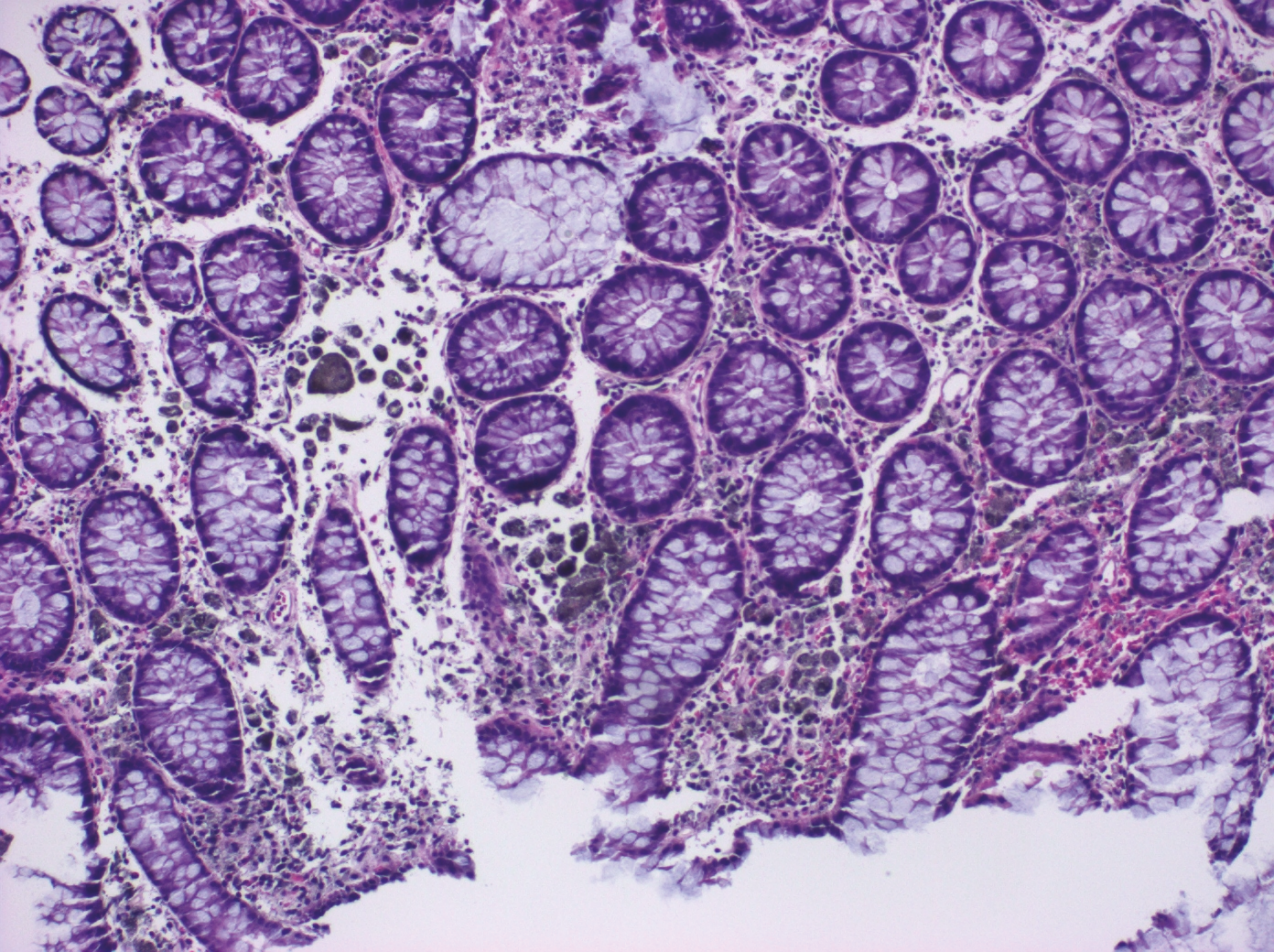
Colonic mucosa demonstrates infiltration of the lamina propria by numerous cells decorated with dark grey pigment, along with some larger giant cells with finely pigmented cytoplasm. (H&E, 200x)

Herein, we report a rare case of ‘true’ colonic melanosis in a young female who underwent colonoscopy for suspected Intussusception. Colonoscopy revealed patchy black pigmentation in the ascending, transverse, descending colon and rectum (Figure 1). Tissue biopsy revealed black-grey pigment that stains with Melan A rather than the typical lipofuscin (2) pigment (Figure 2), commonly seen in patients with melanosis coli because of laxative use (3). The pigment was present inside the atypical cells amidst the inflammatory backdrop. These atypical cells stain with SOX10 and MiTF (4) (Figure 3), which is indicative of origin from neural crest cells. The cells showed a high N/C ratio with prominent nucleoli, but they did not cluster or form mass lesion (Figure 4). Given the deposition of pigment in atypical cells instead of epithelial cells, this is likely a variant of neurocristopathy. The histology was not consistent with any malignant process in our case and a 6-month follow-up revealed similar persistent pigmentation without any suspicion for malignancy.
